# Long-Term Chemical Solubility of 2.3Y-TZP Dental Ceramics

**DOI:** 10.3390/jfb16100374

**Published:** 2025-10-08

**Authors:** Lidija Ćurković, Sanja Štefančić, Irena Žmak, Vilko Mandić, Ivana Gabelica, Ketij Mehulić

**Affiliations:** 1 Department of Materials, Faculty of Mechanical Engineering and Naval Architecture, University of Zagreb, Ivana Lučića 5, 10000 Zagreb, Croatia; ivana.gabelica@fsb.unizg.hr; 2 MarisaDENT, Dugi dol 25, 10000 Zagreb, Croatia; info@marisadent.hr; 3 Department of Inorganic Chemical Technology and Non-Metals, Faculty of Chemical Engineering and Technology, University of Zagreb, Trg Marka Marulića 19, 10000 Zagreb, Croatia; vmandic@fkit.unizg.hr; 4 Department of Fixed Prosthodontics, School of Dental Medicine, University of Zagreb, Gundulićeva 5, 10000 Zagreb, Croatia; mehulic@sfzg.hr

**Keywords:** Y-TZP ceramics, dental ceramics, chemical solubility, chemical stability, dissolution rate constant, ISO 6872:2024

## Abstract

In this study, the chemical solubility (stability) of yttria-partially stabilized zirconia (2.3Y-TZP) dental ceramics, both glazed (Group 2) and non-glazed samples (Group 1), was evaluated using a modified testing protocol based on ISO 6872:2024. Chemical stability was assessed by measuring ion release with inductively coupled plasma mass spectrometry (ICP-MS) and by analyzing phase composition with X-ray diffraction (XRD). While ISO 6872 prescribes chemical stability testing in a 4 wt.% aqueous acetic acid solution at 80 °C for 16 h, the exposure duration in this study was extended to 768 h (32 days) to allow a more accurate determination of long-term solubility behavior. Additionally, the surface roughness parameters (*R*_a_, *R*_max_, *R*_z_, *S_a_*, *S_q_*) were analyzed and evaluated before and after solubility testing. Kinetic analysis revealed that degradation followed a near-parabolic rate law, with power-law exponents of *n* = 2.261 for Group 1 and *n* = 1.935 for Group 2. The corresponding dissolution rate constants were 3.85 × 10^−5^ µg^n^·cm^−2n^·h^−1^ for Group 1 and 132.3 µg^n^·cm^−2n^·h^−1^ for Group 2. XRD results indicated that the long exposure to acetic acid induced a partial phase transformation of zirconia from the tetragonal to the monoclinic phase. Under prolonged acetic exposure, the glaze layer on 2.3Y-TZP exhibited significantly higher dissolution, whereas the zirconia (polished, unglazed) showed low ion release. The temporal change in the total amount of dissolved ions was statistically analyzed for Group 1 and Group 2. The samples showed a strong correlation, but ANOVA confirmed significant differences between them.

## 1. Introduction

Porcelain-fused-to-metal (PFM) restorations continue to be the most popular and successful choice for fixed partial dentures, with low failure rates of about 8–10% over 10 years [1]. However, zirconia ceramics have gained significant attention as structural and biomedical materials in restorative dentistry due to their excellent mechanical properties, chemical resistance, high fracture toughness, and biocompatibility [2]. Zirconia ceramics also display favorable aesthetic qualities, as PFM can cause gingival margin graying due to metal show-through [1]. Full-metal crowns are often seen as the gold standard in dentistry because they have the highest mechanical resistance and remarkable biocompatibility, but their main drawback is aesthetics. Also, the increasing cost of precious metals has limited the use of full metal restorations [3].

The selection of zirconia as a component for dental prosthesis is also driven by its conservative tooth reduction, minimal impact on opposing dentition wear, and reduced risk of veneer chipping [4]. Yttria-partially stabilized zirconia (Y-TZP) crowns often allow for more conservative tooth preparation than metal or PFM restorations because they do not require additional space for veneering porcelain. Zirconia has greater strength than PFM crowns, requiring less tooth reduction; e.g., a PFM crown with 1.5 mm of reduction has similar fracture strength to zirconia crowns with just 1 mm of reduction [3]. The degree of tooth reduction is also influenced by clinical factors such as occlusal clearance, aesthetic requirements, and preparation design [5,6].

Various stabilizing oxides, such as Y_2_O_3_, CeO_2_, CaO, MgO, Yb_2_O_3_, La_2_O_3_, etc., are incorporated to retain the tetragonal or cubic phases at ambient conditions. 3 mol% yttria-stabilized tetragonal zirconia polycrystalline (3Y-TZP) ceramics represent the strongest and toughest yttria-stabilized zirconia polycrystalline ceramics (YSZ), commonly used for fixed dental prosthesis frameworks [7,8]. It is typically slightly white and opaque, and can reach full densification with a fine-grained microstructure, offering high wear resistance, fracture toughness, and exceptional bending strength [9,10,11].

Some 3Y-TZP also contains a small amount of alumina (Al_2_O_3_) particles in its composition, which makes the zirconia slightly less opaque. Alumina enhances powder densification and lowers the sintering temperature [12]. However, this formula of YSZ is susceptible to low-temperature degradation (LTD), also known as hydrothermal aging, when exposed to aqueous medium at human body temperature. During low-temperature degradation, due to spontaneous tetragonal to monoclinic (t→m) transformation of zirconia grains and volume expansion of 3–4%, microcracks form [13].

Generally, phase transformation induces surface roughness and reduces mechanical strength, with the magnitude of this effect depending on composition, processing, and the aging protocol. For example, when 3Y-TZP (standard 3 mol% yttria-stabilized zirconia with 0.25% alumina) and 12Ce-TZP were hydrothermally treated in steam at 134 °C and 3 bar to evoke LTD, biaxial flexural strength was reduced from 1740 MPa to 1169 MPa (after 128 h) and from 495 MPa to 482 MPa, respectively. On the other hand, adding 20 wt.% alumina to 3Y-TZP resulted in an increase from 1093 MPa to 1378 MPa (after 128 h) [14].

Glazing dental ceramics creates a translucent, glossy surface that mimics natural enamel, improving aesthetics. By enhancing optical qualities such as translucency and gloss, glazing helps restorations blend more seamlessly with surrounding teeth [15]. Glazing helps reduce plaque buildup and resistance to extrinsic staining by food or tobacco. Glazing offers a standard, quick, and reliable way to achieve a smooth finish [16]. Specific coloring agents are applied to the surface of the zirconia, often in multiple layers, to achieve the desired shades and translucency [17]. However, glazed zirconia demonstrates lower hardness and wear resistance compared to unglazed polished zirconia [18]. Prior reports indicate that surface finish can influence antagonist behavior: several studies have observed greater antagonist enamel wear against glazed compared with polished zirconia, although outcomes vary with testing protocols and conditions [19].

Dissolution of dental ceramics occurs in the oral cavity due to various chemical or mechanical influences. Dental materials are exposed to a range of pH values in the oral cavity, from highly acidic (due to gastric reflux) to slightly basic (resulting from the ingestion of various liquids and foods).

Monolithic zirconia is highly advised for patients with metal allergies because it does not include a metal framework like porcelain-fused-to-metal (PFM), thereby decreasing the risk of toxic reactions within hard tissue, soft tissue inflammation around the restoration, or hypersensitivity [20]. The temperature also varies depending on whether hot or cold liquids are ingested [21,22]. Therefore, it is of high importance to assess the chemical stability of dental ceramics in various environments to produce long-lasting and stable dental restorations.

The chemical solubility of dental ceramics is usually assessed using the test method outlined in ISO 6872:2024, Dentistry-Ceramic materials, which requires the immersion of specimens with known surface area in 4% acetic acid at a temperature of 80 °C for 16 h [23]. Five percent hydrochloric acid (HCl) can be used to assess the chemical solubility of dental ceramics exposed to gastric acid in patients with gastroesophageal reflux disease and bulimia nervosa, with zirconia and lithium disilicate ceramics being more resistant compared to leucite glass ceramic, feldspathic porcelain, and resin matrix ceramic [24].

The standard method, as specified in ISO 6872:2024, only considers the total surface area (more than 30 cm^2^) but not the morphology or geometry, which may impact test reproducibility [25]. It was demonstrated that cubic specimens were more susceptible to damage due to the handling required by the ISO standard than spherical specimens.

Numerous studies have examined the mechanical [26,27,28] and optical [29] properties, as well as the chemical solubility of zirconia [21,24,25]. However, no data are currently available on the dissolution kinetics of ZrO_2_ dental ceramics. Therefore, an extended solubility test of 2.3Y-TZP dental ceramics (32 days) in 4% acetic acid was performed in this work. Two specimen types were tested: polished unglazed 2.3Y-TZP and polished 2.3Y-TZP with a glazed layer. Chemical degradation was assessed by ion release analysis using high-resolution ICP-MS and phase analysis by XRD. The results were interpreted using kinetic models to quantify dissolution and phase transformation. We hypothesized that (i) glazed 2.3Y-TZP would exhibit higher solubility than polished 2.3Y-TZP, and (ii) extended immersion would provide additional insight into dissolution kinetics.

## 2. Materials and Methods

### 2.1. Characterization of Yttria Partially Stabilized Zirconia (2.3Y-TZP) Dental Ceramics

Two groups of 2.3Y-TZP dental ceramic samples (*n* = 10 per group), stabilized with 2.3 mol.% Y_2_O_3_, i.e., 4.1 wt.% Y_2_O_3_, were prepared. The first group (Group 1) consisted of sintered, polished, and non-glazed specimens. The second group (Group 2) comprised sintered, polished specimens that were fully glazed on all surfaces to simulate the finishing process used in the fabrication of monolithic crowns. Samples of 2.3Y-TZP dental ceramics (both non-glazed and glazed) with the brand name BruxZir Zirconia were provided by Glidewell Laboratories (Newport Beach, CA, USA) as square plates, 10 mm × 10 mm × 2 mm, made via a sintering regime employed in the production of ceramic restorations at Glidewell Laboratories. The glaze was standard and not polished post-firing to preserve the as-glazed surface for chemical-solubility testing. Glidewell Laboratories did not disclose the chemical composition and process parameters for the glaze. Table 1 summarizes the chemical composition of the investigated dental ceramics as provided by the manufacturer of Zirconia Block #18658. A similar study on gastric acidic challenge to the surface topography of monolithic zirconia dental material with the same composition, brand name, and manufacturer was previously conducted by Sulaiman et al. [29].

Based on its Y_2_O_3_ content, the investigated Y-TZP ceramic is designated as 2.3Y-TZP. The phase composition of the 2.3Y-TZP dental ceramics was determined by X-ray diffraction, XRD (Shimadzu XRD6000, Shimadzu Corporation, Kyoto, Japan) with CuKα radiation. A step size of 0.02 degrees between 10° and 80° 2θ, and a counting time of 0.6 s, were used under an accelerating voltage of 40 kV and a current of 30 mA.

The morphology of the prepared sintered samples was analyzed using a scanning electron microscope (SEM) (Tescan Vega TS5136LS, Prague, Czech Republic), in secondary electron (SE) mode, with an acceleration of 30 kV and a magnification of 15,000×. Before the SEM analysis, the polished sample of 2.3Y-TZP dental ceramic was thermally etched at 1480 °C for 12 min as per ASTM Standard E112-96 [30].

The surface roughness parameters (*R*_a_, *R*_max_, *R*_z_) of each sample were measured in five spots using the Perthometer S8P 4.51 with Gaussian data filtering (Feinprüf, Göttingen, Germany). For Group 1, the cutoff length (*λ_c_*) was 0.08 mm and the evaluation length (*L_e_*) was 0.4 mm. For Group 2, *λ_c_*/*L_e_* were 0.25/1.25 mm before acid exposure and 0.8/4.0 mm after acid exposure. Three main roughness parameters were obtained from this test: *R_a_*—the average roughness, *R_z_*—the 10-point average of the profile, and *R_max_*—the maximum between peak and valley.

Moreover, for the evaluation of the surface roughness, Atomic Force Microscopy (AFM) was performed using a Nanosurf CoreAFM (Nanosurf AG, Liestal, Switzerland) device at room temperature in non-contact mode (dynamic mode), employing an aluminum-coated silicon probe (Tap190Al-G, BudgetSensors Innovative Solutions Bulgaria Ltd., Sofia, Bulgaria) with a tip radius of 10 nm and a vibration frequency of 190 kHz. Scans were performed on an area of 10 × 10 µm, with an acquisition time of 0.78 s. The images were processed using CoreAFM Control Software 3.10.5. Two roughness parameters were obtained from this test: *S_a_*—arithmetical mean height (areal average roughness) and *S_q_*—root mean square height relative to the mean plane over the measured area.

### 2.2. Monitoring of the Chemical Solubility of the 2.3Y-TZP Dental Ceramics

Sample groups of 2.3Y-TZP were measured for size, and then washed in distilled water using an ultrasonic bath (1510 DTH, Electron Microscopy Sciences, Hatfield, PA, USA) to remove any contaminants. They were subsequently dried at 150 °C. The mass of each sample was measured using an analytical balance (Ohaus Analytical Plus, OHAUS Corporation, Parsippany, NJ, USA) to an accuracy of ±0.01 mg, and the surface area was calculated. Each sample was completely immersed in 10 mL of 4 wt.% aqueous CH_3_COOH solution in individual polypropylene (PP) tubes, sealed and placed in a thermostatic shaker (Innova 4080 Incubator-Shaker, Herisau, Switzerland), operating at 80 °C and 200 rpm.

The dissolution tests were conducted at a temperature of 80 °C from 16 h to 32 days (768 h). Acetic acid presents an acidic challenge relevant to the oral environment, and elevated temperatures accelerate dissolution processes. The exposure time was extended to 32 days to estimate long-term dissolution kinetics and dissolution trends. ISO 6872 specifies that chemical solubility testing should be performed using specimens with a minimum total surface area of 30 cm^2^ immersed in 100 mL of 4 wt.% acetic acid at 80 °C. Still, it does not prescribe a strict surface-area-to-volume (SA/V) ratio of the solution. To ensure reproducibility, we calculated and reported the SA/V ratio applied in this study. Each specimen had a surface area of 2.8 cm^2^ and was immersed in 10 mL of solution, resulting in a SA/V ratio of 0.28 cm^2^·mL^−1^. This ratio was close to the minimum SA/V of 0.3 cm^2^·mL^−1^ proposed in the standard. Measurements were conducted after 16 h, 8 days (192 h), 16 days (384 h), and 32 days (768 h) of immersion. Only 10 samples were used, and the acid was replaced after each time period. After each interval, the samples were washed with distilled water in an ultrasonic bath, dried, and weighed. For each exposure condition, ten replicates were tested simultaneously (*n* = 10). All obtained results are presented as mean value ± standard deviation.

The acid solution was collected for each interval, and concentrations of Al^3+^, Na^+^, Ca^2+^, K^+^, Si^4+^, Fe^3+^, Zn^2+^, Mg^2+^, Sr^2+^, Ba^2+^, Y^3+^, and Zr^4+^ ions released into the testing solution were determined using high-resolution inductively coupled plasma mass spectrometry (HR-ICP-MS; Thermo Fisher Scientific, Waltham, MA, USA). The results are reported as the amount of ions (M^n+^) released per unit surface area of the tested 2.3Y-TZP dental ceramic samples. After the time had elapsed, the samples were washed with distilled water in an ultrasonic bath, dried, and weighed.

Statistical analyses were performed using the Analyse-it software package (trial copy, Version 6.16.2, Ltd., Leeds, UK) and MS Excel to evaluate the temporal change in the total concentration of dissolved ions for Group 1 and Group 2. Descriptive statistics and comparative tests were applied, including a paired *t*-test and a one-way ANOVA. These methods were employed to determine whether the differences between the samples were statistically significant and to identify any potential systematic biases in their behavior over time.

An ANOVA was conducted to evaluate the effects of the power exponent (*n*) and the parabolic dissolution rate constant (*K*_p_). The *F*-values and corresponding *p*-values were used to determine statistical significance at the 95% confidence level (*p* < 0.05). The relative contribution of each factor to the overall variability was quantified through the distribution of the sum of squares, allowing comparison of their importance within the system.

## 3. Results

### 3.1. Structural and Morphological Characterization of Yttria Partially Stabilized Zirconia (2.3Y-TZP) Dental Ceramics

**Figure 1:**
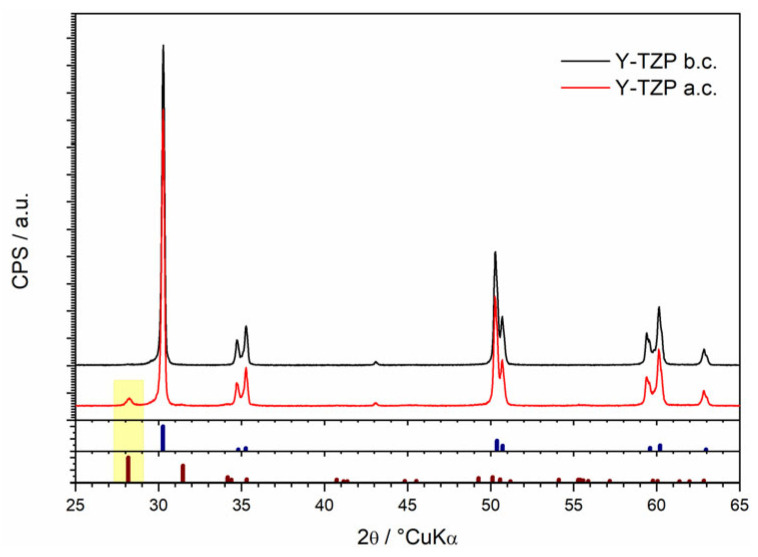
Comparison of diffraction patterns of non-glazed 2.3Y-TZP dental ceramics before (Y-TZP b.c.) and after (Y-TZP a.c.) solubility test (blue and brown vertical bars represent the standard diffraction peaks of tetragonal (ICDD PDF No. 50-1089) and monoclinic (ICDD PDF No. 37-1484) ZrO_2_, respectively; yellow box represents monoclinic phase).

To investigate the correlation between the dissolution process and phase transformations in 2.3Y-TZP dental ceramics, samples were analyzed by X-ray diffraction (XRD) before and after exposure to the testing medium (Figure 1). The yellow highlight denotes the presence of the monoclinic phase, and the bottom two lines present the standard reference spectra for each ZrO_2_ phase.

**Figure 2:**
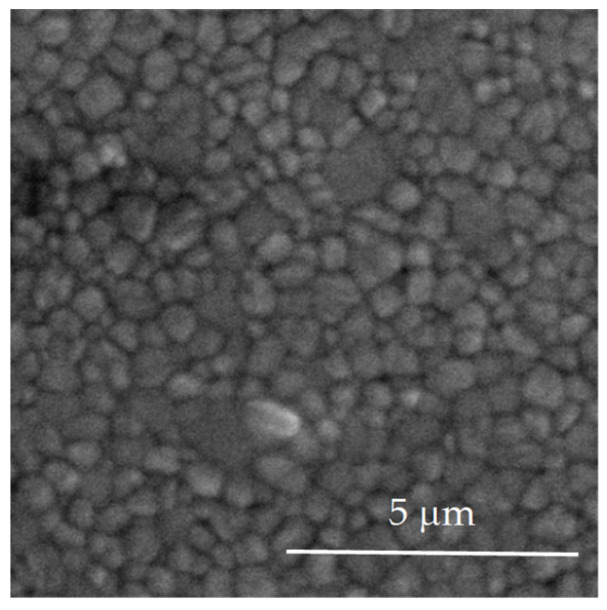
Polished and thermally etched surface image of the 2.3Y-TZP dental ceramics (thermal etching at 1480 °C, 12 min).

Figure 2 shows the SEM micrograph of the polished and thermally etched surface of 2.3Y-TZP dental ceramics (Group 1).

The main roughness parameter results are summarized in Table 2. Before acid exposure, glazed 2.3Y-TZP showed a higher profilometric roughness than polished, unglazed 2.3Y-TZP (*R_a_* 0.27 ± 0.021 μm vs. 0.005 ± 0.001 μm), reflecting microscale waviness of the glassy overlayer.

**Figure 3:**
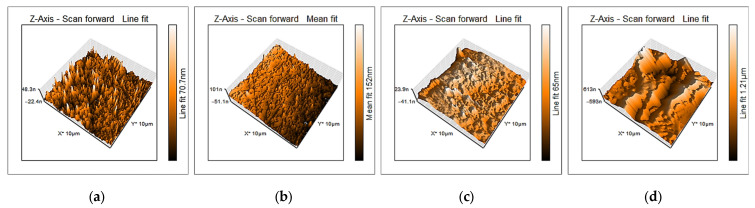
AFM 3D-micrographs of 2.3Y-TZP different surfaces: (**a**) non-glazed before acid exposure; (**b**) non-glazed after acid exposure; (**c**) glazed before acid exposure; (**d**) glazed after acid exposure.

Surface morphology of 2.3Y-TZP (Group 1 and Group 2) obtained by AFM is shown in Figure 3, and the corresponding roughness parameters *S_a_* and *S_q_* are in Table 3. 3D-AFM pictures indicated both surfaces were nanoscale-smooth before surface dissolution, consistent with profilometry results and optical observation (the samples appear glossy despite having a higher profilometry roughness parameter; micrometer-scale *R_a_*).

### 3.2. Amount of Ions Released in Testing Solution from Yttria Partially Stabilized Zirconia (2.3Y-TZP) Dental Ceramics

**Figure 4:**
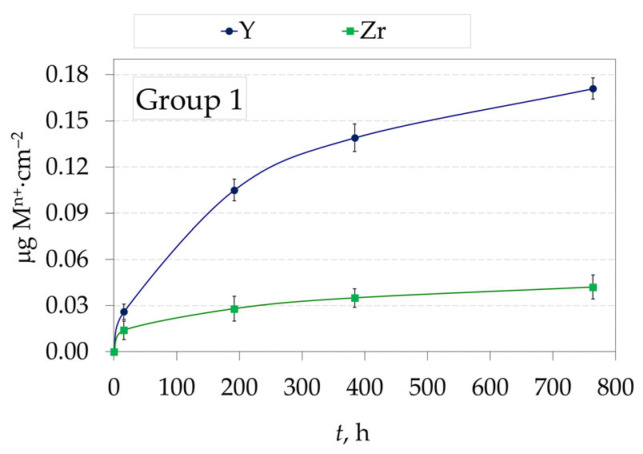
Amount of eluted metal ions (M^n+^), i.e., Y^4+^ and Zr^4+^, from non-glazed 2.3Y-TZP dental ceramics (Group 1) in 4 wt.% CH_3_COOH solution as a function of immersion time at 80 °C (mean value and standard deviation).

Figure 4 shows the amount of eluted Y^4+^ and Zr^4+^ ions from non-glazed 2.3Y-TZP dental ceramics (Group 1) and the time of immersion in the 4 wt.% CH_3_COOH solution at 80 °C.

**Figure 5:**
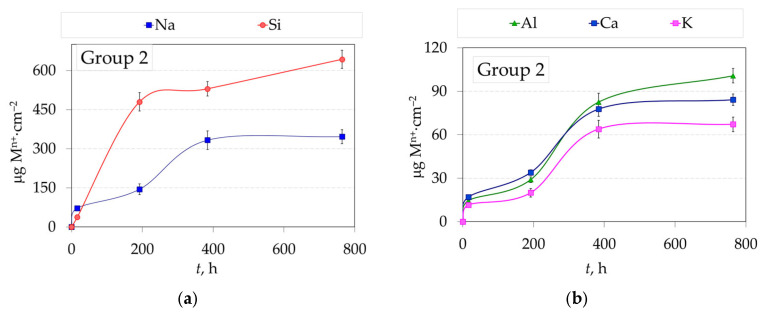

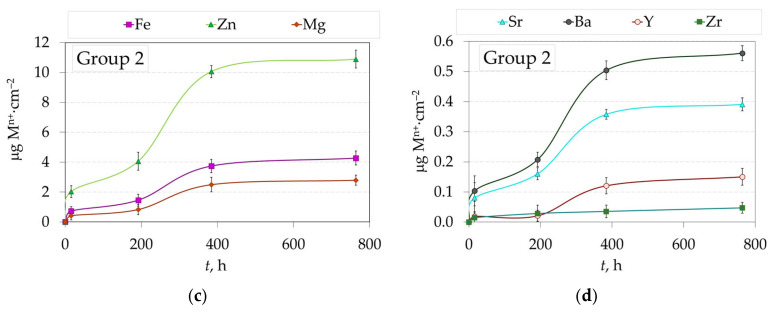
The amount of different eluted metal ions, M^n+^ (**a**) Na^+^, Si^4+^; (**b**) Al^3+^, Ca^2+^, K^+^, (**c**) Fe^3+^, Zn^2+^, Mg^2+^; (**d**) Sr^2+^, Ba^2+^, Y^3+^ and Zr^4+^ ions from Group 2 in a 4 wt. % CH_3_COOH solution as a function of immersion time at 80 °C (mean value and standard deviation).

Figure 5 shows the relationship between the amount of eluted Na^+^ and Si^4+^ (Figure 5a), Al^3+^, Ca^2+^ and K^+^ (Figure 5b), Fe^3+^, Zn^2+^, Mg^2+^ (Figure 5c), Sr^2+^, Ba^2+^, Y^3+^ and Zr^4+^ ions (Figure 5d) from 2.3Y-TZP dental ceramics Group 2 (sintered, polished and glazed) and the time of immersion in the 4 wt.% CH_3_COOH solution at 80 °C.

**Figure 6:**
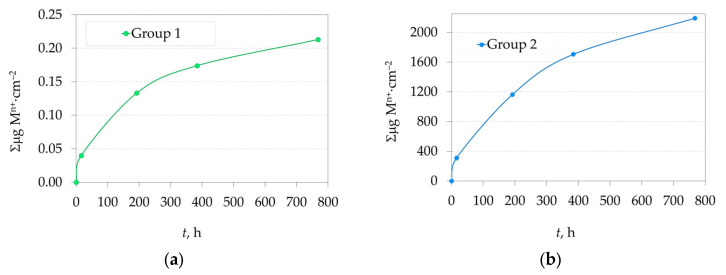
Total eluted metal ions (M^n+^) from 2.3Y-TZP dental ceramics: (**a**) Group 1; (**b**) Group 2 after immersion in 4 wt.% CH_3_COOH solution as a function of immersion time at 80 °C (note the difference in scale between the groups).

The total amounts of eluted ions from 2.3Y-TZP dental ceramics (Group 1 and Group 1) after immersion in 4 wt.% CH_3_COOH solution as a function of immersion time at 80 °C are presented in Figure 6a,b.

The paired *t*-test was used to compare the means of Group 1 and Group 2 at the same time points for the total amount of eluted metal ions (M^n+^). The *t*-value was large in magnitude (−141.45), and the *p*-value was very small (7.79 × 10^−7^), i.e., *p* < 0.05, indicating a statistically significant difference between the values of Group 1 and Group 2. One-way ANOVA confirmed that the means of Group 1 and Group 2 differ significantly: The *F*-value was very high (251), and the *p*-value was well below 0.05 (4.01 × 10^−6^), confirming the *t*-test result that there is a significant difference between Group 1 and Group 2. The Pearson correlation coefficient of 0.999, with a highly significant *p*-value (0.00099), indicates a strong positive linear relationship between Group 1 and Group 2. Although the samples are strongly correlated, Bland–Altman analysis revealed that the samples differ systematically, despite moving in the same direction, as the mean difference (bias) was not close to zero: rather, it was high (−9.11).

### 3.3. Dissolution Rate of Yttria Partially Stabilized Zirconia (2.3Y-TZP) Dental Ceramics in CH_3_COOH

The measurement of eluted ions was used to investigate the dissolution kinetics. The variation in dissolution rate over time may follow linear, parabolic, logarithmic, or other rate laws. The following equation was applied to determine which rate law best describes the experimental data:

(1) $${\left(\sum \frac{m(M^{n+})}{A}\right)}^{n}=K_{p}\cdot t$$

where $m\;(M^{n+})$ stands for the mass of the overall amount of eluted ions (Al^3+^, Ca^2+^, Fe^3+^, Mg^2+^, Na^+^), µg; *A* for specific surface, cm^2^; *n* for the power exponent; *K*_p_ for dissolution rate constant, µg^n^·cm^−2n^·h^−1^; and *t* for the time of the ceramic exposure to the testing media, h.

**Figure 7:**
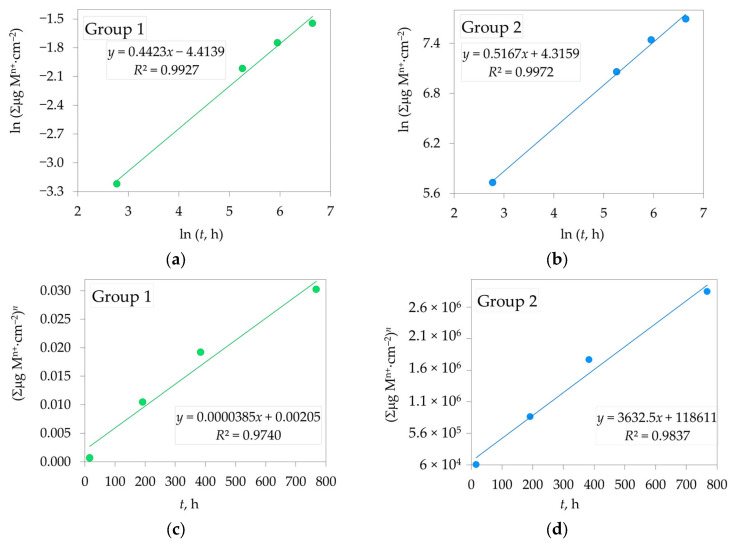
Determination of the power exponent *n* for: (**a**) Group 1; (**b**) Group 2; Determination of the dissolution rate constants *K*_p_ for: (**c**) Group 1; (**d**) Group 2 of 2.3Y-TZP dental ceramics in the 4 wt.% CH_3_COOH solution at 80 °C (M^n+^, metal ions).

The power exponent *n* was determined from the linear plot of ln (Σμg (M^n+^·cm^−2^) versus ln *t* (Figure 7a,b), with *n* calculated from the slope of the line. The dissolution rate constants *K*ₚ were obtained from the slope of the linear plot of (Σμg(M^n+^·cm^−2^)*^n^* versus *t* (Figure 7c,d). The calculated values of the power exponent (*n*) and the dissolution rate constants (*K*_p_, µg^n^·cm^−2n^·h^−1^) for Groups 1 and 2 are summarized in Table 4. High correlation coefficients (*R*^2^) were observed for both samples.

The ANOVA for the obtained model results shows that all examined factors have a statistically significant effect on the response variable (*p* < 0.05), as indicated by the consistently high *F*-values (Table 5). The distribution of the sum of squares also highlights the relative importance of these factors in explaining the observed variability, with the largest contribution (4.1 *×* 10^12^) clearly dominating the variance structure. Group 1’s regression model is highly significant, with strong explanatory power and minimal error. Group 2’s model fits well, nearly perfectly explaining the data with high significance. Both models have high *R*^2^ values, low residual errors (e.g., in modeling *K*_p_ 0.01219 for Group 1 and 0.006611 for Group 2), and significant *p*-values, indicating that ln(*t*, h) is a strong predictor for both groups.

## 4. Discussion

In the samples exposed to 4 wt.% CH_3_COOH at 80 °C (after dissolution, 2.3Y-TZP a.c.), the tetragonal ZrO_2_ phase (t-ZrO_2_) remained the dominant crystalline phase. However, a small amount of monoclinic ZrO_2_ (m-ZrO_2_) (ICDD 37-1484) was also detected (highlighted in yellow in Figure 1). The semi-quantitative content of the m-Zirconia has been calculated at 0.7 wt.% Via the Toraya equation [31]; a minor transformation from the tetragonal to the monoclinic phase of ZrO_2_ occurred during acid exposure.

The average grain size, determined according to ASTM Standard E112-96 (2004), was 425 ± 137 nm (Figure 2).

The data presented in Table 2 demonstrate distinct differences in the surface roughness of Non-Glazed Vs. Glazed 2.3Y-TZP specimens following exposure to 4 wt.% acetic acid at 80 °C for 32 days. For the non-glazed 2.3Y-TZP (Group 1), all three measures of roughness (*R_a_*, *R_max_*, *R_z_*) showed no appreciable variation between the initial and post-immersion measurements (*R_a_* = 0.005 µm), indicating a high degree of chemical stability. The glazed 2.3Y-TZP (Group 2) exhibited a substantial increase in all roughness parameters, with *R*_a_ rising from 0.27 µm to 2.75 µm, accompanied by corresponding large increases in *R_max_* and *R_z_*. This pronounced surface degradation indicates that the glaze layer is considerably more susceptible to acetic acid attack than the unglazed zirconia.

Due to low values of roughness parameters (*R_a_*, *R_max_*, *R_z_*) for Group 1 (Table 2), roughness was additionally analysed by AFM. AFM has confirmed that the glazed samples appeared much more susceptible to acid exposure, with a visibly altered surface and a roughness increase (*S_a_*, *S_q_*), as obtained by AFM measurements, of more than an order of magnitude (Table 3 and Figure 3). Glazing improves optical gloss and uniformity, whereas polishing yields the lowest profilometric roughness; the obtained data align with this distinction and explain why the unexposed glazed surfaces appear optically smooth yet measure rougher than polished zirconia at the microscale.

Both zirconia Groups exhibited minimal release of Zr^4+^ and Y^3+^ ions, indicating excellent chemical stability of the testing ceramic samples under the tested conditions (Figure 4 and Figure 5d). Despite the relatively low yttria content (4.1 wt.%, 2.3 mol%) in 2.3Y-TZP ceramics, the concentration of Y^3+^ in the leachate exceeded that of Zr^4+^ at every sampling time for both groups, indicating higher aqueous solubility of yttria under the tested acidic conditions (4 wt.% CH_3_COOH, 80 °C). Glazed 2.3Y-TZP (Group 2) released the following ions (Si^4+^, Na^+^, Al^3+^, Ca^2+^, K^+^, Zn^2+^, Fe^3+^, Mg^2+^, Ba^2+^, Sr^2+^, Y^3+^, and Zr^4+^), with Si^4+^ being dominant. After 32 days, the release order of eluted ions was Si^4+^ > Na^+^ > Al^3+^ > Ca^2+^ > K^+^ > Zn^2+^ > Fe^3+^ > Mg^2+^ > Ba^2+^ > Sr^2+^ > Y^3+^ > Zr^4+^. The dissolution mechanism in both cases was consistent with dissolution by simple dissociation. After testing for 32 days, the amount of dissolved Y^3+^ was slightly less in glazed samples (Group 2, 0.150 µg·cm^−2^) compared to non-glazed samples (Group 1, 0.171 µg·cm^−2^), due to the phase dissolution of glazed samples and the simultaneous dissolution of zirconia by diffusion of the acetic media through the glaze layer. The amount of dissolved Zr^4+^ after testing for 32 days showed no significant difference between the two groups (0.042 µg·cm^−2^ and 0.047 µg·cm^−2^) for Group 1 and Group 2, respectively.

Typical dental glazes are glasses of silica with a primary composition of sodium and alumina. Baria or strontia may be included for color, change of refractive index, or radiopacity, with strontium favored due to a superior safety profile in oral applications [32,33].

The overall ion release represents the cumulative sum of all individual ions leached from the 2.3Y-TZP ceramics into the acetic acid solution throughout the experiment (Figure 6). In general, the dissolution increased with immersion time for both 2.3Y-TZP samples. These findings suggest that Group 2, which contains a glaze layer, is more susceptible to dissolution.

The non-glazed zirconia samples complied with the ISO 6872:2024 requirement, as their chemical solubility after 16 h was 0.04 µg·cm^−2^, well below the specified limit of 100 µg·cm^−2^. Even after 32 days of exposure, the solubility remained below this threshold, with a value of 0.21 µg·cm^−2^. The glazed samples also met the ISO 6872:2024 requirements after 16 h, with a chemical solubility of 308 µg·cm^−2^ compared to the permissible limit of 2000 µg·cm^−2^. After immersion up to 384 h (16 days), the solubility increased to 1704 µg·cm^−2^, and to 2192 µg·cm^−2^ after 32 days. Based on the dissolution kinetics, we estimate that the threshold of 2000 µg·cm^−2^ was reached after approximately 24 days of immersion, under these accelerated conditions. The relationship between this dissolution and in vivo lifetime has not been established.

The power exponent (*n*) was 2.261 and 1.935 for Group 1 and Group 2, respectively, which means that the dissolution kinetics of 2.3Y-TZP ceramics in the 4 wt.% CH_3_COOH aqueous solution at 80 °C follows the near-parabolic law (Table 3, Figure 7a,b), which means that the dissolution rate is decreasing over time.

The dissolution rate constant (*K*_p_, Table 3, Figure 7c,d) for Group 2 (glazed 2.3Y-TZP) was significantly higher at approximately 3600 compared to the value without glaze (3.85 × 10^−5^, Group 1). This study did not include instrumental optical measurements, but a qualitative assessment was made. After acidic exposure, a loss of surface sheen was observed in the glazed specimens (Group 2), whereas the polished 2.3Y-TZP showed only subtle changes (Group 1). Clinically, these findings support prioritizing high polish finishing on zirconia surfaces exposed to the oral environment over glazed zirconia.

In the framework of this study, the hypotheses that (i) glazed 2.3Y-TZP would exhibit higher solubility than polished 2.3Y-TZP, and (ii) extended immersion would provide additional insights into dissolution kinetics, were confirmed.

In future studies, we plan to include mechanical and optical property testing before and after acid exposure.

## 5. Conclusions

XRD confirmed that the bulk of the tested zirconia was tetragonal, with only a minor monoclinic fraction after acid exposure. Uncoated (non-glazed) 2.3Y-TZP exhibited excellent chemical stability throughout the test period: ion release of Zr^4+^ and Y^3+^ remained very low (with Y^3+^ slightly higher than Zr^4+^), surface roughness changes were minimal, and the cumulative chemical solubility at 32 days was 0.21 µg·cm^−2^—orders of magnitude below the ISO 6872 threshold of 100 µg·cm^−2^. These findings indicate that the zirconia substrate itself is highly resistant to dissolution under the examined conditions.

The glassy glaze layer was markedly more susceptible to degradation, showing progressive dissolution and pronounced topographical alteration. Although glazed 2.3Y-TZP met ISO requirements in the standard 16 h assessment, solubility increased continuously with time, surpassing 2000 µg·cm^−2^ after ~24 days and reaching 2192 µg·cm^−2^ at 32 days.

Taken together, the results emphasize that the zirconia remains chemically stable. In contrast, the glaze, which is an optional finishing layer used primarily for aesthetic purposes and not an integral part of the main restoration, exhibits limited durability in prolonged exposure to acidic conditions. Mechanical polishing of zirconia, therefore, represents a robust alternative finish when long-term chemical stability is prioritized.

## Figures and Tables

**Table 1:** Chemical composition of the 2.3Y-TZP dental ceramics expressed as weight and molar percent (wt.% and mol%) (wt.% is reproduced with permission from Ref. [28]. 2014 Elsevier.)

Element	Y_2_O_3_	HfO_2_	Al_2_O_3_	SiO_2_	Fe_2_O_3_	Na_2_O	ZrO_2_
wt.%	4.1	4.0	0.34	<0.01	<0.01	<0.01	balance (91.56)
*M*, g⋅mol^−1^	225.81	210.49	101.96				123.22
*n*, mol	0.0182	0.0190	0.0033	--	--	--	0.74
mol.%	2.32	2.33	0.42	--	--	--	94.81

**Table 2:** Values of the roughness parameters obtained by Profilometer (mean value and standard deviation) of Group 1 (non-glazed 2.3Y-TZP) and Group 2 (glazed 2.3Y-TZP) before and after 32 days of immersion in 4 wt.% CH_3_COOH solution at 80 °C.

2.3Y-TZP	*R_a_*, µm		*R_max_*, µm		*R_z_*, µm	
	Before Acid Exposure	After Acid Exposure	Before Acid Exposure	After Acid Exposure	Before Acid Exposure	After Acid Exposure
Group 1	0.005 ± 0.001	0.005 ± 0.001	0.046 ± 0.003	0.053 ± 0.004	0.043 ± 0.003	0.047 ± 0.005
Group 2	0.27 ± 0.021	2.75 ± 0.31	1.33 ± 0.042	22.23 ± 3.05	1.02 ± 0.043	19.98 ± 1.52

**Table 3:** Roughness parameters obtained by AFM analysis for Group 1 (non-glazed 2.3Y-TZP) and for Group 2 (glazed 2.3Y-TZP).

2.3Y-TZP	*S_a_*, nm	*S_q_*, nm
Group 1: Before acid exposure	7.01	9.35
Group 1: After acid exposure	8.97	10.24
Group 2: Before acid exposure	5.67	7.73
Group 2: After acid exposure	140.38	182.25

**Table 4:** Values of the power exponent (*n*) and parabolic dissolution rate constant (*K*_p_) for Group 1 (non-glazed 2.3Y-TZP) and for Group 2 (glazed 2.3Y-TZP), mean value and standard deviation.

2.3Y-TZP	*n*	*R* ^**2**^	*K*_p_, µg^**n**^·cm^**−2n**^·h^**−1**^	*R* ^**2**^
Group 1	2.261 ± 0.004	0.99	3.85 × 10^−5^ ± 4.45 × 10^−6^	0.97
Group 2	1.935 ± 0.015	0.99	3632.5 ± 330.7	0.98

**Table 5:** ANOVA results for the power exponent (*n*) and the parabolic dissolution rate constant (*K*p) for Group 1 (non-glazed 2.3Y-TZP) and Group 2 (glazed 2.3Y-TZP).

Model	2.3Y-TZP	Sum of Squares	*F*-Value	*p*-Value
*n*	Group 1	1.672	274.23	0.0036
	Group 2	2.280	689.65	0.0014
*K*_p_	Group 1	0.00046	74.87	0.0131
	Group 2	4.1 × 10^12^	120.66	0.0082

## Data Availability

The original contributions presented in the study are included in the article; further inquiries can be directed to the corresponding author.

## Abbreviations

The following abbreviations are used in this manuscript:

12Ce-TZP	12 mol% ceria-stabilized tetragonal zirconia polycrystal
2.3Y-TZP	2.3 mol% yttria-stabilized tetragonal zirconia polycrystal
3Y-TZP	3 mol% yttria-stabilized tetragonal zirconia polycrystal
AFM	Atomic force microscopy
c	Cubic phase
Ce-TZP	Ceria-stabilized tetragonal zirconia polycrystal
CNC	Computer numerical control machining
HR-ICP-MS	High-resolution inductively coupled plasma mass spectrometry
ICP-MS	Inductively coupled plasma mass spectrometry
LTD	Low-temperature degradation
m	Monoclinic phase
PFM	Porcelain-fused-to-metal
SA/V	surface-area-to-volume
SEM	Scanning electron microscope
t	Tetragonal phase
XRD	X-ray diffraction
Y-TZP	Yttria partially stabilized zirconia polycrystal
YSZ	Yttria-stabilized zirconia polycrystal
